# AI-Based Analysis of Ziehl–Neelsen-Stained Sputum Smears for *Mycobacterium tuberculosis* as a Screening Method for Active Tuberculosis

**DOI:** 10.3390/life14111418

**Published:** 2024-11-03

**Authors:** Arief Budi Witarto, Bogdan Ceachi, Cristiana Popp, Sabina Zurac, Ioana Cristina Daha, Flora Eka Sari, Nirawan Putranto, Satria Pratama, Benyamin P. Octavianus, Luciana Nichita, Julian Gerald Dcruz, Cristian Mogodici, Mirela Cioplea, Liana Sticlaru, Mihai Busca, Oana Stefan, Irina Tudor, Carmen Dumitru, Alexandra Vilaia, Alexandra Bastian, Gheorghita Jugulete, Gyula Laszlo Fekete, Petronel Mustatea

**Affiliations:** 1 Medical Biodefense Research Center, Faculty of Medicine and Health Sciences, Indonesia Defense University, Kawasan IPSC Sentul, Sukahati, Kecamatan Citeureup, Kabupaten Bogor 16810, Indonesia; witarto@gmail.com (A.B.W.); floraeka68@gmail.com (F.E.S.); nirmataku@yahoo.co.id (N.P.); tspratama@gmail.com (S.P.); 2 Department of Pathology, Colentina University Hospital, 21 Stefan Cel Mare Str., Sector 2, 020125 Bucharest, Romania; ceachi.bogdan@gmail.com (B.C.); brigaela@yahoo.com (C.P.); luciana.nichita@umfcd.ro (L.N.); cristian.mogodici@zaya.ai (C.M.); mirelacioplea@yahoo.com (M.C.); liana_ro2004@yahoo.com (L.S.); thanatogenesis@gmail.com (M.B.); oana.stefan93@yahoo.com (O.S.); irinafrincu@yahoo.com (I.T.); carmendumitru2004@yahoo.com (C.D.); alexandra.bastian@umfcd.ro (A.B.); petronel.mustatea@umfcd.ro (P.M.); 3 Zaya Artificial Intelligence SRL, 9A Stefan Cel Mare Str., 077190 Voluntari, Romania; julian.dcruz@zaya.ai; 4 Faculty of Automatic Control and Computer Science, National University of Science and Technology Politehnica Bucharest, 313 Splaiul Independenţei, Sector 6, 060042 Bucharest, Romania; 5 Department of Pathology, University of Medicine and Pharmacy Carol Davila, 37 Dionisie Lupu Str., Sector 1, 020021 Bucharest, Romania; alexandra.vilaia@gmail.com; 6 Department of Cardiology, Colentina University Hospital, 21 Stefan Cel Mare Str., Sector 2, 020125 Bucharest, Romania; 7 Department of Cardiology, University of Medicine and Pharmacy Carol Davila, 37 Dionisie Lupu Str., Sector 1, 020021 Bucharest, Romania; 8 Badan Kesira Gerindra, Jl. Harsono RM No. 54, Ragunan, Pasar Minggu, Jakarta Selatan 12160, Indonesia; octavianusbenny@yahoo.com; 9 Department of Infectious Diseases, National Institute for Infectious Diseases “Prof. Dr. Matei Balș”, 1 Dr. Calistrat Grozovici Str., Sector 2, 021105 Bucharest, Romania; gheorghita.jugulete@umfcd.ro; 10 Department of Infectious Diseases, University of Medicine and Pharmacy Carol Davila, 37 Dionisie Lupu Str., Sector 1, 020021 Bucharest, Romania; 11 Department of Dermatology, George Emil Palade University of Medicine, Pharmacy, Science and Technology of Targu Mures, 38 Gh. Marinescu Str., 540142 Targu Mures, Romania; dermafek@yahoo.com; 12 Department of Surgery, Ion Cantacuzino Clinical Hospital, 011437 Bucharest, Romania; 13 Department of Surgery, University of Medicine and Pharmacy Carol Davila, 37 Dionisie Lupu Str., Sector 1, 020021 Bucharest, Romania

**Keywords:** artificial intelligence, tuberculosis, *Mycobacterium tuberculosis*, Ziehl–Neelsen, screening

## Abstract

Tuberculosis is the primary cause of death due to infection in the world. Identification of *Mycobacterium tuberculosis* in sputum is a diagnostic test, which can be used in screening programs—especially in countries with a high incidence of tuberculosis—to identify and treat those persons with the highest risk of disseminating the infection. We previously developed an algorithm which is able to automatically detect mycobacteria on tissue; in particular, our algorithm identified acid-fast bacilli on tissue with 100% specificity, 95.65% sensitivity, and 98.33% accuracy. We tested this algorithm on 1059 Ziehl–Neelsen-stained sputum smears to evaluate its results as a possible tool for screening. The results were displayed as a heat map of 32 × 32 pixel patches. Analysis of the positive patches revealed a good specificity (86.84%) and 100% sensitivity for patches with a level of confidence over 90; furthermore, the accuracy remained over 95% for all levels of confidence over 80, except the class (95–100]. The modest specificity is caused by the peculiarities of smears (uneven thickness, dust contamination, lack of coverslip). We will train the algorithm on sputum smears to increase the specificity to over 95%. However, as our algorithm showed no false negatives, it is suitable for screening.

## 1. Introduction

Tuberculosis (TB) is a deadly infectious disease caused by *Mycobacterium tuberculosis*; the TB epidemic in the 18th−19th centuries in Western Europe reached a high yearly mortality rate of up to 800–1000 deaths per 100,000 people [1]. In 2022, the TB mortality reached 16.4 deaths per 100,000 people (1.3 million deaths), representing the eighth-highest cause of death worldwide and the leading cause of death due to an infectious disease (except for 2020 and 2021, when it was outpaced by COVID-19) [2,3]. There was a general decrease in new and relapsed TB cases in 2020 and 2021, due to the COVID-19 pandemic (a global reduction of 18.5% in 2020 compared with 2019 and 9.6% in 2021 compared with 2019); in 2022, the TB incidence rate was similar to the incidence rate in 2019 (7.451 million new and relapsed TB cases in 2022, similar to 7.12 million cases in 2019) [4].

TB is present in every geographic location; however, almost half of the cases are present in Southeast Asia (46%), with Indonesia accounting for 10% of the total TB cases worldwide. The total TB incidence in 2022 in Indonesia was 1.06 million cases with a rate of 385 new cases per 100,000 people and a mortality of 140,700 people due to TB per year (TB mortality rate of 51.4 per 100,000 people) [5]; TB was the fourth most frequent cause of death in Indonesia in 2019 after stroke, ischemic cardiac disease, and diabetes mellitus [6]. Indonesia experienced an increase in the incidence of TB in 2022 of more than 5% over 2015; the TB incidence diminished considerably in 2020 and 2021, due to the impossibility for many patients to seek and receive proper medical care, related to the lack of medical assistance during the COVID-19 pandemic. However, it significantly recovered in 2022; preserving this recovery is mandatory for the future [7].

A reduction in the TB incidence rate (compared with the 2015 baseline) is one of the three milestones of the WHO End TB Strategy; the milestone aims to diminish the incidence by 20% in 2020 and 50% in 2025 (compared with the 2015 incidence). The reduction reached 18.5% in 2020; however, the reduction was mainly caused by the COVID-19 pandemic. The 2022 TB incidence has been reduced by only 8.7%, quite far from the 50% milestone established for 2025. Efforts should be made to improve the diagnosis and treatment of TB patients, especially of those with positive sputum; these people actively spread the disease, putting their contacts at a much greater risk of contamination with *Mycobacterium tuberculosis* and developing the disease than contacts of TB patients with positive cultures but negative sputum [8]. National screening programs focused on the detection of mycobacteria (acid-fast bacilli, AFB) in sputum are a logical solution.

*Mycobacterium tuberculosis* is a small bacillus identifiable on smears by thorough examination with 40× or even 100× magnification of specially stained slides; the most commonly used special stain for mycobacteria is the Ziehl–Neelsen (ZN) stain. A sputum smear is quite large—usually 4–5 cm^2^—requiring at least 30 min and sometimes hours for examination when the smear is negative or paucibacillary [9]. One easy solution is to use AI-based algorithms to identify AFB-positive cases. We previously developed an algorithm which is able to automatically detect *Mycobacterium tuberculosis* on tissue stained with ZN; our algorithm was designed to identify AFB on tissue, not on sputum, and had a specificity of 100%, a sensitivity of 95.65%, and an accuracy of 98.33%, performing as well as the best pathologist in a test against human examiners. We decided to test this algorithm on sputum smears in order to evaluate its results as a possible tool for screening.

## 2. Materials and Methods

We selected 1309 cases of sputum smears (1265 positive and 44 negative).

The positive smears (1265 cases) were taken during diagnostic procedures from patients admitted to two hospitals: RSUD Pasar Rebo, Jakarta, and RSAU Dr Hassan Toto, Bogor, Indonesia. All the smears were diagnosed as positive based on the microscopic identification of acid-fast bacilli; the microscopic examination (and, thus, labeling them as positive) was performed by certified pathologists (human examiners). Further, the diagnosis of tuberculosis was confirmed by microbiologic cultures and/or molecular tests (Xpert MTB/RIF performed on GeneXpert platform). In total, 44 negative sputum smears were taken from healthy medical personnel from the Department of Pathology, Colentina University Hospital, Bucharest, Romania; these smears were confirmed as negative by two separate pathologists (human examiners), and no discrepancies were noted.

The status of the smears (positive versus negative) was confirmed.

The study was conducted in accordance with the Declaration of Helsinki and approved by the Institutional Ethics Committee of Colentina University Hospital (protocol code 13/17.11.2021) and The Medical Research Ethics Committee of the Faculty of Medicine and Health Sciences, Indonesia Defense University (protocol code no. 2023-12-005/26.11.2023).

The smears were prepared using the protocol [10,11] detailed in Table 1.

**Figure 1:**
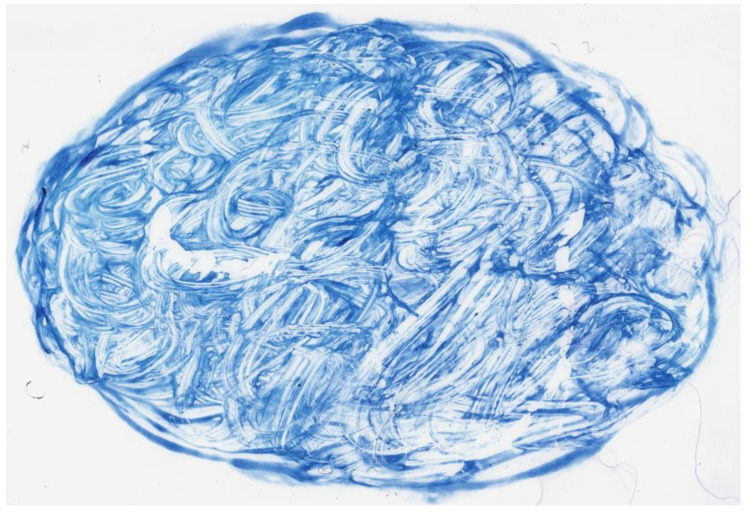
Sputum smear stained with Ziehl–Neelsen stain; the biological material is spread as an ellipse of 27.8 × 18.06 mm. ZN × 0.16.

The samples were of similar sizes, in the range of 2.5–3 cm/1.5–2.2 cm; one example is shown in Figure 1.

The smears were scanned using automatic scanners, Leica Aperio AT2 and Leica Aperio GT450 (Leica Biosystems, Deer Park, IL, USA). In total, 1309 whole-slide images (WSIs) in “.svs” format were obtained. We screened each WSI by opening it with Aperio ImageScope Pathology Slide Viewing Software version 12.4.6.5003 (Leica Biosystems, Deer Park, IL, USA). The scans were performed in one plane; no Z-stacked images were used. In total, 1289 smears were scanned using the 40× scanning mode, and 20 smears were scanned using the 20× scanning mode. Each WSI was examined by two pathologists to establish the quality of the image. Smears unproperly scanned, scanned with objective 20×, too thick, or with staining defects on more than 90% of the surface of the smear were discarded from the study group.

**Figure 2:**
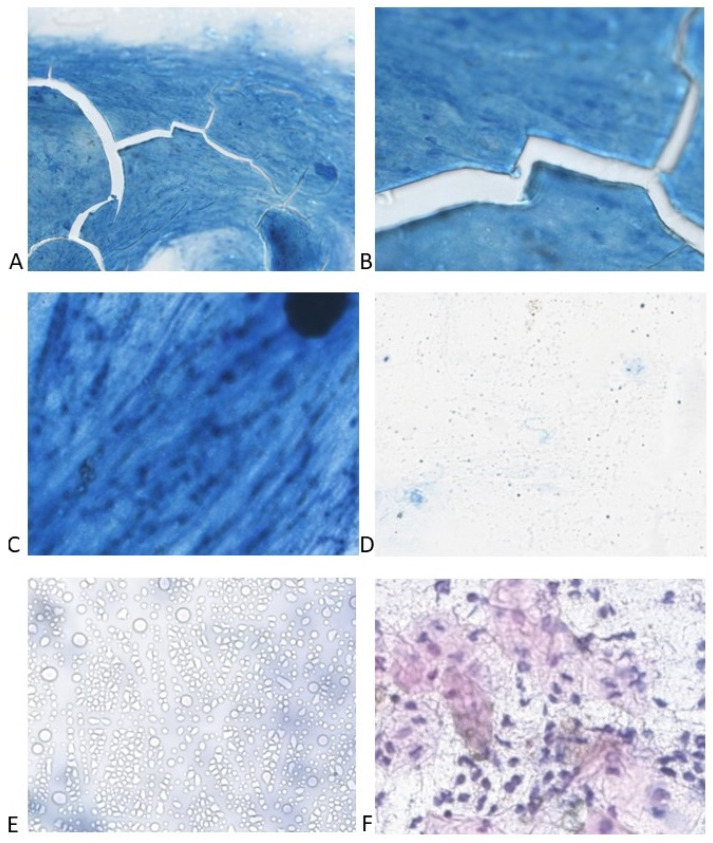
Examples of unsuitable smears. (**A**) Smear burned during ZN staining. Several cracks are easily noticeable. ZN × 100. (**B**) Detail of the previous smear; no identifiable structures are present. ZN × 400. (**C**) Smear too thick; some structures can be seen, but they are not recognizable. ZN × 400. (**D**) Smear too thin; almost no material is present. ZN × 400. (**E**) Smear full of artifacts. ZN × 400. (**F**) Smear with staining defect; epithelial cells are pink, and large pink precipitates are present elsewhere on the smear. ZN × 400.

In Figure 2, several examples of unsuitable WSIs are presented.

All the remaining WSIs were submitted to automatic analysis for *Mycobacterium tuberculosis* presence using our in-house developed algorithm [12]. In our previous work, technically, we used a deep convolutional neural network (CNN)—specifically, a customized version of RegNetX4. This network is designed for efficient and scalable image analysis tasks. We made several modifications to the base architecture. We reduced the kernel size in the initial layer to better capture the small features of *Mycobacterium tuberculosis*, as the morphology of the bacillus can be effectively captured using smaller convolution filters, such as 3 × 3 or 5 × 5. This helps to focus on the fine details necessary for accurate identification [12].

We adjusted the number of convolutions to maintain an appropriate field of view, given the small size of the target objects, ensuring that the network could effectively process features without losing important details. This adjustment allowed the network to retain the focus on individual bacilli while covering a sufficient contextual area [12].

We incorporated dilated convolutions to improve the detection of objects with varying shapes, which is particularly helpful in distinguishing the different morphologies of *Mycobacterium tuberculosis* and overcoming the challenges related to overlapping cells or artifacts in ZN-stained slides [12].

We used reflection padding to make the network more robust to variations in the placement of the bacilli within the image patches, minimizing the boundary artifacts that could otherwise mislead the network during feature extraction [12].

The dataset used for the training of that algorithm was annotated using the Cytomine application (Cytomine Corporation SA, Liège, Belgium). The model development was completed using PyTorch v1.11 as the deep learning framework [12].

We trained our model in a distributed fashion using parameter replicas for each graphics processing unit (GPU) and gradient averaging before broadcasting the parameter updates. We used a batch size of about 2048 per GPU, with positive and negative examples roughly evenly split to mitigate the severe class imbalance. We experimented with various proportions (positive vs. negative) moving from 25–75% to 75–25%, using 5% increments to determine the optimal balance. The training process was limited to a maximum of 100 million samples seen (including augmented patches). The AdaBound optimizer was used, with the step size α provided by a linear warm-up cosine scheduler with periodic restarts, which helped achieve stable convergence. To enhance the model’s generalization capabilities, we applied extensive image augmentations, including random rotations, shifts, brightness, contrast adjustments, and saturation changes. Depending on the WSI size and the number of CPU threads used, the baseline processing pipeline managed to process a WSI in 5 to 15 min [12].

As previously presented, we developed the algorithm based on highly supervised training on a manually annotated dataset of ZN-stained histopathologic sections of tissue. No additional training on sputum smears was performed. The results on smears were displayed as a heat map of 32 × 32 pixel patches in batches with different levels of confidence with an increment of 5: (95–100], (90–95], (85–90], (80–85], (75–80], (70–75], (65–70], (60–65], (55–60], (50–55], (45–50], (40–45], (35–40], (30–35], (25–30], (20–25], (15–20], (10–15], (5–10], (0–5]. Each patch could include one or more bacilli, either single or in clusters; since the presence of one bacillus is sufficient to label the smear as positive, we did not differentiate between isolated bacilli or clusters of them. On each WSI, after applying all the filters for the abovementioned levels of confidence (i.e., confidence (0–100]), significant areas of the smears remained unlabeled (zero confidence for bacilli presence). For further analysis, we examined patches with levels of confidence over 50 (at least a 50% chance to identify a bacillus in that patch).

We analyzed the results of the algorithm versus the ground truth (microscopic analysis of the smear by a pathologist), as detailed in Table 2.

To evaluate the performance of our algorithm on ZN-stained WSIs, we used sensitivity, specificity, and accuracy as the performance metrics.

## 3. Results

Our initial group of samples consisted of 1309 cases of sputum smears, including 1265 positive and 44 negative smears. Most of the smears were in the range of 2.5–3 cm/1.5–2 cm (Figure 1). After visual analysis of the WSIs with an Aperio ImageScope, we selected 1021 positive WSIs (80.71%) and 38 negative WSIs (86.36%). All the remaining 1059 WSIs were single-layer WSIs scanned in 40× scanning mode, well-scanned, well-stained, and with enough biological material on them. The numbers of excluded cases are listed in Table 3, along with the cause for exclusion.

All the remaining 1059 slides were analyzed using the previously developed in-house algorithm for identifying AFB. As expected, the algorithm returned suggestions of positive patches with various levels of confidence for every WSI, irrespective of their status (positive or negative). We accepted the findings with levels of confidence over 50. The positive WSIs had an extremely variable number of probable positive patches, from 68 to 527,526. To superimpose the results of the algorithm on the IUATLD-WHO scoring system, we calculated the approximate number of 100× high power fields (HPFs) present on an ellipse with radii of 3 × 2 cm, which is approximately 3000 (3000 HPF on a smear). We transformed the number of AFB counted by a pathologist on 100 HPF into the number of bacilli on the whole slide and made it equivalent with the IUATLD-WHO as follows: negative: 0 positive patches on the WSI; scanty: less than 300 bacilli on the WSI (10 bacilli on a “length” of 100 HPF equal to 300 positive patches on WSI); 1+: less than 3000 bacilli on the WSI (100 bacilli on a “length” of 100 HPF equal to 3000 positive patches on WSI); 2+: less than 30,000 bacilli on the WSI (10 bacilli on a HPF equal to 30,000 positive patches on WSI); 3+: over 30,000 bacilli on the WSI. The results are presented in Table 4.

**Figure 3:**
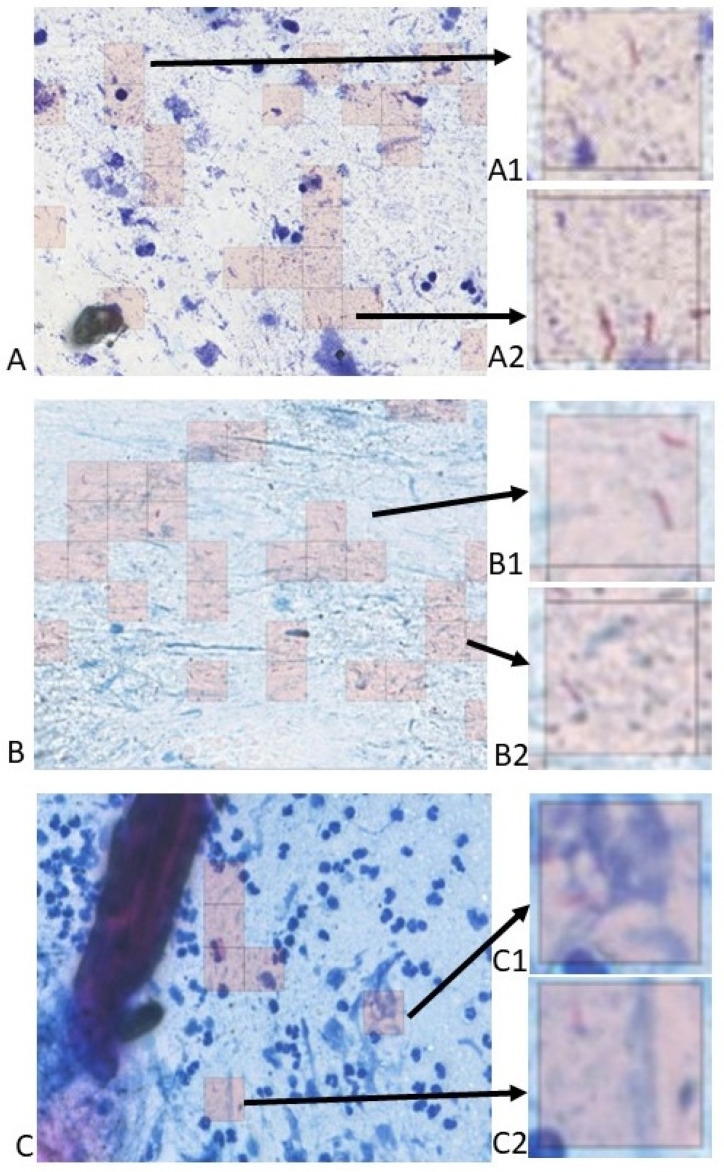
Numerous bacilli are present in all pictures. However, artifacts are present (precipitates in (**A**), stain deposits in (**C**)). (**A**) ZN × 400. A1 and A2 (details) ZN × 2000; (**B**) ZN × 400. B1 and B2 (details) ZN × 2000; (**C**) ZN × 400. C1 and C2 (details) ZN × 2000.

**Figure 4:**
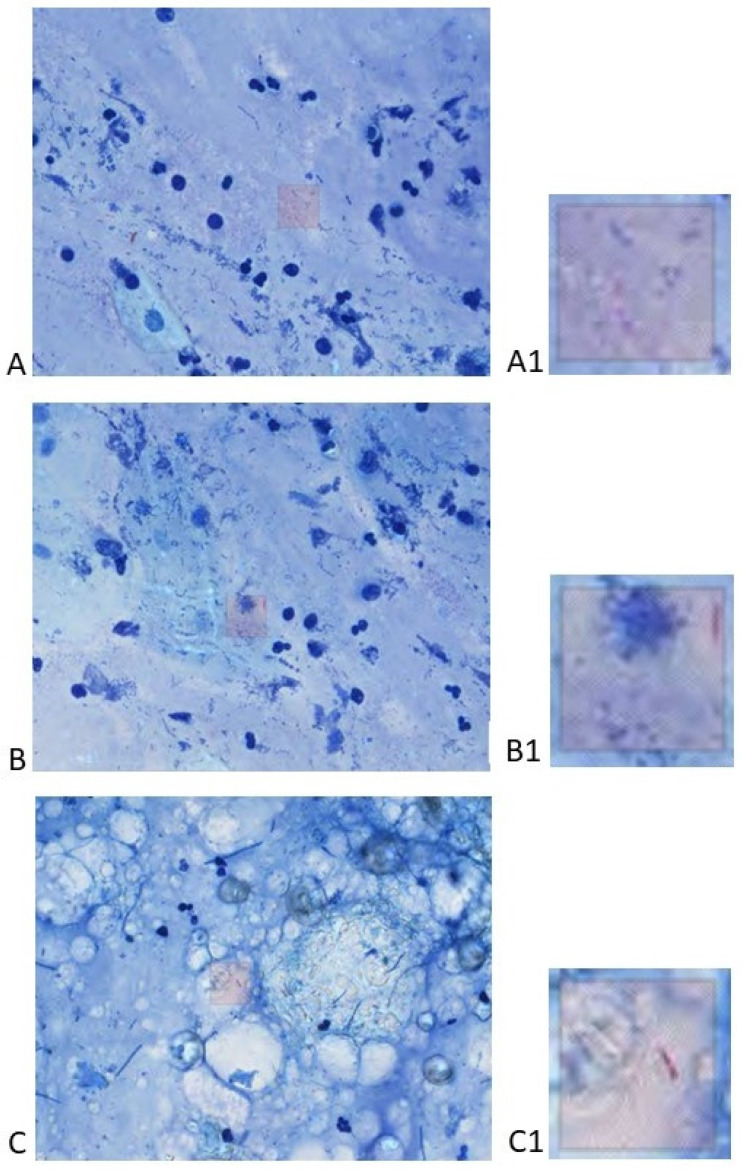
Paucibacillary smears. Note that there is a large variability in the cellularity of the background with few inflammatory cells. (**A**) ZN × 400. A1 (detail) ZN × 2000; (**B**) ZN × 400. B1 (detail) ZN × 2000; (**C**) ZN × 400. C1 (detail) ZN × 2000.

The analysis of the positive patches reveals a very good specificity for a high level of confidence, as can be observed in Figure 3 and Figure 4. The algorithm also labeled artifacts, especially thin long red structures; however, the general level of confidence for this confusion was low. We analyzed the performance metrics for several levels of confidence (Table 3). The highest interval had a good specificity (92.11%) but a very low sensitivity (18.81%) (Table 5); when patches with a level of confidence (90–95] were added, the specificity decreased as expected (from 92.11% to 86.84%), but the sensitivity reached 100% (Table 6). Adding patches with a level of confidence (85–90], the specificity decreased to 47.37% (Table 7), and a new addition of patches with a level of confidence (80–85] caused the specificity to decrease to zero (Table 8). Interestingly, the accuracy remained over 95% for all levels of confidence over 80, except the class (95–100]; obviously, the accuracy was maintained over 95% due to the imbalance of the dataset. Adding further levels of confidence did not change the performance metrics.

In order to balance our dataset, we calculated the performance metrics for a 38 positive/38 negative dataset by arbitrarily selecting 38 positive WSIs. As all the positive cases had positive patches with a level of confidence (90–100] (true positive 1021 cases: Table 6), each set of 38 positive WSIs had the same results (Table 9), no matter how we selected them. For a balanced smear set of 76 cases with an equal number of positive and negative cases, the sensitivity was 100%, the specificity was 86.84%, and the accuracy was 93.42% for patches with a level of confidence over 90.

## 4. Discussion

To date, there have been several attempts to develop algorithms for ZN-stained sputum analysis [13], most of them based on pictures (i.e., separate images taken from smears) [14,15,16,17,18,19,20]. Few studies have used WSIs to develop their algorithms [21], and some used randomly selected images from a previously scanned smear [22,23]. The number of cases used for developing the algorithm varied from fifty cases to several thousand. Seventeen studies reported an accuracy over 0.9519 [24,25,26,27,28,29,30,31,32,33,34,35,36,37,38,39], all but two with a sensitivity over 0.95 (the remaining two with a sensitivity of 0.947 [38] and 0.949 [34]). None of them were tested in clinical settings against human examiners.

We developed an algorithm able to automatically identify AFB on a section of tissue using Ziehl–Neelsen stain [12]. Although the algorithm was designed on tissue slides, the results on the smears were very good. When the cut-off for the level of confidence was set to 90, the sensitivity was 100% (“no positive patients are left behind”); the specificity was quite modest (86.84%), meaning that 13.16% of the results were false positives. There are significant differences between the sputum and tissue appearance. The tissue is sectioned in even slices (usually 3 microns thick) and, after staining, it is placed between a slide and a coverslip, keeping it clean. Sputum is spread on the slide usually with a disposable loop, and the result is inherently uneven in thickness; the smear is not covered with a coverslip and is easily contaminated with dust or other particles. As scanning is performed with a 40× objective lens, using coverslips could be a solution to diminish the number of artifacts. We intend to train our algorithm on sputum smears to increase the specificity to over 95%.

There are several lines of action for significantly reducing the TB incidence, one of the most important being identification of people with TB in the population. Several screening methods can be applied, but the most effective one is that which is able to actively identify the people who spread bacilli by coughing or merely speaking. These people have AFB in their sputum; thus, a screening method able to easily identify bacilli in the smears is the logical answer. Preparing sputum smears is easy and cheap, and does not require sophisticated equipment or highly trained personnel. Examination of the ZN-stained smears is expensive and time-consuming, requiring qualified examiners; in fact, using sputum smear examination for screening is difficult due to the long time required for the microscopic analysis of each specimen and the shortage of pathologists and trained personnel. The current development of AI-based methods of analysis in histopathology will change this situation and allow the use of sputum smear analysis for AFB in screening programs.

A problem discussed in several papers is which method is the most appropriate to use in screening to identify possible TB patients.

Mass miniature radiography (MMR) identifies various types of pulmonary lesions, some of which are caused by TB; it was used in the past for screening the general population. However, there are several drawbacks: the radiological appearance is not specific for TB; people with radiologic lesions are not necessarily contagious; the method cannot be applied to all people (pregnant women for example) and incurs risks due to X-ray exposure; the sensitivity of the method is quite low and has no possibility of identifying positive sputum smear cases that occur between two rounds of examination; and, as it requires equipment, infrastructure, and highly trained personnel, the associated costs are quite high [40].

Interferon gamma release assay (IGRA) is a blood test measuring the level of the IFN-gamma response of the T lymphocytes from harvested blood to peptides that simulate antigens derived from *Mycobacterium tuberculosis* [41]. These antigens are ESAT-6 and CFP-10, present in the *Mycobacterium tuberculosis* complex (*M. tuberculosis*, *M. bovis*, *M. africanum*, *M. microti*, and *M. canetti*) and absent from Bacillus Calmette–Guerin used in vaccination programs and from almost all non-tuberculous mycobacteria strains (except *M. marinum*, *M. kansasii*, and *M. szulgai*). Several in vitro diagnostic tests are commercially available, either an enzyme-linked immunospot (ELISPOT) or enzyme-linked immunosorbent assay (ELISA) [42,43]. All of these identify all *Mycobacterium tuberculosis*-infected people, no matter whether they have a latent or active disease. Since these people represent roughly 25% of the global population [44], most likely in higher proportion in areas with high prevalence of TB, the test is not suitable for screening—the large number of positive subjects to be further tested will overwhelm the health system. In addition, the cost of the IGRA test per se is quite high.

A bacteriological culture represents the gold standard of diagnosing TB, as it identifies active cases of tuberculosis with live bacilli in the sputum. It has high specificity and sensitivity and offers supplementary data concerning drug susceptibility but requires specific infrastructure and highly trained personnel and has significant costs. There is a considerable risk of contamination during various manipulations and subsequent false positive results. In addition, another drawback is the very long period until the results are ready (up to 3 months) [7].

Molecular tests such as real-time polymerase chain reaction (rtPCR) Xpert MTB/RIF performed on GeneXpert platform have the advantage of identifying *Mycobacterium tuberculosis* and the mutations associated with the detection of drug resistance (rifampicin resistance). The results are obtained quite rapidly (less than 2 h) but require dedicated equipment, special infrastructure, highly trained personnel, and, thus, considerable costs. Even in the case of subsidies, the costs are significant [45]. Contamination is also a problem.

Sputum smear examination is an older method still used in low- and middle-income countries for diagnosing TB. As already stated, it has several advantages, being a simple method which is completely safe for patients (it can be used without risks both in children, pregnant women, or debilitated persons). Smear preparation is cheap and does not require special equipment or environmental conditions. The microscopic examination provides results in a short time (usually 30 min). The most important advantage consists in identification of those people with clinical TB forms with the highest risk of transmitting the pathogen. The main drawback is the need for specialized trained personnel to examine the slides. The shortage of pathologists and trained technicians has a subsequent consequence on the current recommended protocol of the microscopic examination—the examiner is required to examine “one length” of approximately 2 cm of the smear; in paucibacillary smears, this approach may render numerous false negative results; moreover, ocular and nervous fatigue will hamper the results, even in case of experienced pathologists [46].

From the arguments presented above, it is obvious that an AI-based sputum smear examination mitigates the limitations of the sputum smear examination as a method for screening. Moreover, it is expected that the sensitivity of the method will improve significantly, since it will identify paucibacillary cases overlooked by a human examiner. The costs will increase slightly due to the inclusion of a slide scanner in a regional center. The results of the screening can be used in diagnosis through confirmation of the positive result provided by the algorithm through the examining of the algorithm’s heat map by a pathologist.

We intend to develop an algorithm which is capable of finding most of the AFB-positive sputum patients in Indonesia, which has the second-highest TB burden in the world after India (10% of the world’s TB cases in 2022) and the fourth in terms of prevalence (the prevalence of bacteriologically confirmed pulmonary TB in surveys completed between 2007 and 2021 in Indonesia is 759 cases per 100,000 people (95% uncertainty interval 590–961)) [7,47]. Our algorithm was developed based on a dataset consisting of positive and negative patches taken from images of histopathologic sections. We tested it on sputum, and it identified bacilli on each positive case; in addition, it did not identify too many false positive images either on positive or negative smears. We have already started to develop another dataset based on sputum smears to develop an algorithm dedicated to identifying AFB in sputum; we intend to test the algorithm on a more balanced smear set, closer to Indonesian data.

One important problem that should be taken into consideration is that sputum smears have uneven thickness. In daily routine, the pathologist is permanently refocusing the image, a maneuver that is impossible when evaluating single-image plane captures of slides; multilayer WSIs (captured as a z-stack of images scanned along the *z*-axis) offer more details and successfully mitigate the biases induced by using single-layer WSIs especially in paucibacillary cases [48]. However, the resulting images are much larger and more difficult to upload; moreover, the scanning equipment is more expensive and requires more time and more experienced personnel to operate it. We used our algorithm on single-layer WSIs on purpose, in order to test its capacity to detect AFB on routinely prepared sputum smears and scanned with relatively simple scanners. Moreover, when scanning sputum (either by a human examiner or via automatic detection), the identification of one convincing AFB is sufficient to label that smear as positive. Thus, in our opinion, it is not important to identify all the bacilli present in a smear but, instead, to identify the smear as a positive one. The number of bacilli automatically identified by the algorithm in the positive dataset is quite impressive, despite the use of single-layer WSIs (23.02% cases had between 3000 and 30,000 positive patches, equivalent to a 2+ WHO-IUATLD score, while 54.46% cases had over 30,000 positive patches, equivalent to a 3+ WHO-IUATLD score, Table 4).

## 5. Conclusions

Our algorithm, despite being trained and validated on tissue sections, was able to identify *Mycobacterium tuberculosis* on sputum with an accuracy of 0.9342, a specificity of 0.8684, and a sensitivity of 1.00 for a level of confidence over 90. We intend to retrain our algorithm on sputum smear images to increase the specificity. However, even in its current form, our algorithm is suitable for screening, as its results show no false negative cases.

## Figures and Tables

**Table 1:** Preparation of sputum smear.

1. Record the code of the specimen on the frosted end of a new clean slide.
2a. In the case of a non-centrifugated fresh sample of sputum, select purulent areas of the specimen with a stick or a disposable loop.
OR
2b. In the case of a centrifugated (3000× g for 20 min) fresh sample of sputum, re-suspend the pellet and collect the biologic material with a disposable loop.
3. Press the loop/stick on the center of the slide and move it concentrically over an oval area of 2–3 cm/1–2 cm.
4. Air-dry the smear.
5. Fix the slide by passing it over a flame 2–3 times for about 2–3 s each time or by keeping it for two hours on hot plates at 65–75 °C.
6. Stain the slide with Ziehl–Neelsen stain.
6.1. Heat the underside of the slide with an open flame for 5 min after the steam appears. In the case of the intense vaporization of the reagent, add more. Do not let the reagent boil.
6.2 Rinse with tap water until clear; then, drain off the excess.
6.3 Cover the slide with 3% acid–alcohol for 2–3 min; then, drain.
6.4 Rinse with tap water until clear; then, drain off the excess.
6.5 Cover the slide with 0.3% methylene blue for one minute; then, drain.
6.6 Rinse with tap water until clear; then, drain off the excess.
6.7. Air-dry.

**Table 2:** Reporting acid-fast bacilli (AFB) on Ziehl–Neelsen stained smears; WHO-IUATLD grading score.

WHO-IUATLD Score	Brightfield (1000× Magnification: 1 Length = 2 cm = 100 HPF)
Negative	Zero AFB/1 length or 100 HPF
Scanty; report the exact number of AFB	1–9 AFB/1 length or 100 HPF
1+	10–99 AFB/1 length or 100 HPF
2+	1–10 AFB/1 HPF in at least 50 fields
3+	>10 AFB/1 HPF in at least 20 fields

WHO = World Health Organization. IUATLD = International Union Against Tuberculosis and Lung Disease.

**Table 3:** Numbers of cases excluded from the study, separated according to the cause of exclusion.

Cause of Exclusion	Positive Smears		Negative Smears	
	Number	%	Number	%
**Scanning with 20×**	20	1.58%	0	0.00%
**Bad scanning**	89	7.04%	0	0.00%
**Too thick**	18	1.42%	1	2.27%
**Too thin**	29	2.29%	3	6.82%
**Bad staining**	88	6.96%	2	4.55%
**Total**	244	19.29%	6	13.64%

**Table 4:** Number of patches with a level of confidence >50 in positive and negative cases.

Number of Patches with Level of Confidence >50	Positive Smears		Negative Smears	
	Number	%	Number	%
**<300**	12	1.18%	5	13.16%
**300–3000**	218	21.35%	15	39.47%
**3000–30,000**	556	54.46%	17	44.74%
**>30,000**	235	23.02%	1	2.63%
**Total**	1021	100.00%	38	100.00%

**Table 5:** Number of patches with a level of confidence (95–100] in positive and negative cases (1059 cases, imbalanced dataset).

	True	False
**Positive**	192	3
**Negative**	35	829

Specificity: 0.9211; sensitivity: 0.1881; accuracy: 0.2143.

**Table 6:** Number of patches with a level of confidence (90–100] in positive and negative cases (1059 cases, imbalanced dataset).

	True	False
**Positive**	1021	5
**Negative**	33	0

Specificity: 0.8684; sensitivity: 1; accuracy: 0.9953.

**Table 7:** Number of patches with a level of confidence (85–100] in positive and negative cases (1059 cases, imbalanced dataset).

	True	False
**Positive**	1021	20
**Negative**	18	0

Specificity: 0.4737; sensitivity: 1; accuracy: 0.9811.

**Table 8:** Number of patches with a level of confidence (80–100] in positive and negative cases (1059 cases, imbalanced dataset).

	True	False
**Positive**	1021	38
**Negative**	0	0

Specificity: 0; sensitivity: 1; accuracy: 0.9641.

**Table 9:** Number of patches with a level of confidence (90–100] in positive and negative cases (76 cases, balanced dataset).

	True	False
**Positive**	38	5
**Negative**	33	0

Specificity: 0.8684; sensitivity: 1; accuracy: 0.9342.

## Data Availability

The datasets used and/or analyzed during the current study and also tests of the algorithm are available from the corresponding author(s) on reasonable request.
